# Patients Experiencing Unsuccessful Defibrillation From Implantable Cardiac Devices Remain at Elevated Risk Despite Procedural Management

**DOI:** 10.1111/jce.70310

**Published:** 2026-03-08

**Authors:** Nathaniel Christian‐Miller, Muazzum Shah, Kelly Arps, Amrish Deshmukh, Jackson Liang, Ryan Cunnane, Krit Jongnarangsin, Frank Pelosi, Hakan Oral, Michael Ghannam

**Affiliations:** ^1^ Division of Cardiovascular Medicine, Department of Electrophysiology University of Michigan Ann Arbor Michigan USA

## Abstract

**Background:**

Unsuccessful defibrillation therapy in patients with implantable cardiac defibrillators (ICDs) and ventricular tachytherapies may occur due to patient and/or device‐related factors; appropriate management strategies after failed defibrillation therapy have been incompletely described.

**Objectives:**

To report on the management and outcomes of patients with implantable cardiac devices and unsuccessful defibrillation therapies.

**Methods:**

A single‐center cohort of patients with ICDs was examined, patients with unsuccessful ICD therapies were included. Demographic and device features, and survival free from recurrent unsuccessful device therapy was examined among patients undergoing operative vs. non‐operative management.

**Results:**

Among 1449 patients with ICDs, 40 patients (2.8%) were identified with unsuccessful defibrillation therapies (mean age 59 ± 15 years, ejection fraction 29% ± 16%, ischemic cardiomyopathy *n* = 27, 67.5%, secondary prevention device placement *n* = 22, 55%, transvenous ICD *n* = 34, 85%, subcutaneous ICD *n* = 6, 15%). Nonoperative management strategies included device reprogramming (*n* = 8), addition of a class III anti‐arrhythmic (*n* = 7), or conservative therapy (*n *= 5). Operative management included addition of a transvenous lead (*n* = 10), addition of subcutaneous array (*n* = 8), or change in pulse generator (*n* = 2). A single‐coil device was present in 18/20(90%) patients undergoing operative management compared to 10/20 (50%) with non‐operative management (*p* = 0.16). There were no demographic or device differences between the two groups. After 2.4 ± 2.1 years follow up, repeat VT occurred in 22 patients (55%) including 6 patients (15%) with a repeat failed defibrillator therapy. There was no differences in the risk of recurrent failed shocks among patients with operative vs non‐operative management (log rank *p* = 0.14).

**Conclusions:**

Among a large cohort of patients with ICD, the incidence of failed defibrillator therapy was 2.8%. With appropriate patient selection, both operative and non‐operative management led to similar long‐term outcomes; however, the overall incidence of repeat failed defibrillation therapy remained high at 15%.

Cardioversion via implantable cardiac defibrillators (ICD) can terminate life‐threatening ventricular arrhythmias (VA), and has been shown to improve mortality in selected patients [1]. Failure of an ICD to terminate VA (i.e., “failed shocks”) may occur due to patient and/or device‐related factors, or may be due to the probabilistic nature of defibrillation itself [2]. Real‐world data on management strategies and the risk of a repeat failed shocks are lacking. The purpose of this research letter is to describe management approaches to failed shocks and to report on the long‐term recurrence rates.

This was single‐center retrospective evaluation of 1449 patients with ICDs followed from 2017 to 2024, among whom 40 (2.8%) experienced a failed‐shock. Patients were included if there was appropriate detection of VA and appropriate delivery of defibrillation therapy which failed to terminate the arrhythmia. This study was approved by the University of Michigan Institutional Review Board.

The average age was 59 ± 15 years with a mean ejection fraction of 29% ± 16%. Six patients (15%) had left ventricular assist devices, 27 (67.5%) had a ischemic cardiomyopathy, 34 (85%) had transvenous ICDs (single coil n = 28(70%), dual coil *n* = 6(15%)), subcutaneous ICDs *n* = 6(15%). After a failed shock, 14 patients (35%) experienced syncope, 4 patients (10%) required external cardioversion, 3 patients (8%) had spontaneous conversion after multiple failed shocks, and 33 patients (83%) had successful subsequent shocks during the arrhythmia episode.

Twenty patients underwent procedural management, and 20 patients underwent non‐procedural management. Patients with non‐procedural management were mostly deemed to have reversible causes of a failed shock including acute decompensated heart failure (*n* = 10), electrolyte abnormalities (*n* = 2), acute ischemia (*n* = 2), VT storm (*n* = 5), or shock occurring during recovery from LVAD implant (*n* = 1). Nonoperative management strategies included device reprogramming to alternative shock vectors (*n* = 8), addition of a class III anti‐arrhythmic (*n* = 7), or conservative therapy (*n* = 5). Operative management included the addition of a new transvenous right ventricular lead (*n* = 9), addition of an azygous coil (*n* = 1), addition of subcutaneous array (*n* = 8), or change in pulse generator to allow for adjustment of the defibrillation waveform (*n* = 2). All patients underwent successful defibrillation threshold testing and reprogramming with a 5–10J safety margin. There were no complications related to acute management.

After 2.4 ± 2.1 years follow‐up, repeat VA occurred in 22 patients (55%) including 6 patients (15%) with a repeat failed defibrillator therapy (Figure 1). There were no statistically significant differences in the rates of recurrent failed shocks among patients with operative vs. non‐operative management (log rank *p* = 0.14). Management after a failed shock included pulse generator revision, addition of an azygous coil, addition of a subcutaneous array, subcutaneous ICD implantation, addition of sotalol, and device reprogramming. Two/6 patients had repeat VA, one of whom had a successful shock and one with a third incidence of failed shock treated with device reprogramming.

**Figure 1:**
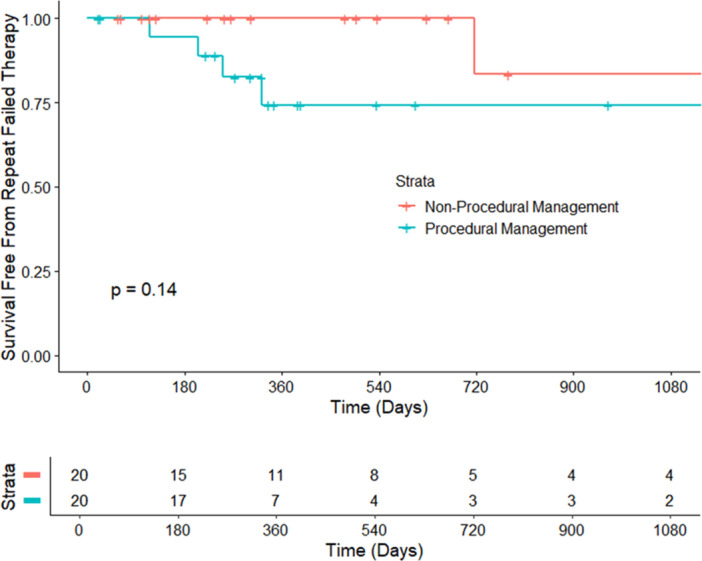
Survival free of repeat unsuccessful defibrillation therapy among patients undergoing procedural and non‐procedural management.

The main findings from this report are that (1) in a contemporary real‐world cohort of ICD patients the rate of failed shock was approximately 0.4%/year and (2) the overall incidence of repeat failed shocks was high at 15% on mid‐term follow‐up up with a trend towards higher rates in patients without clearly reversible causes who underwent procedural management.

To our knowledge, this is the first report to detail the incidence of failed shocks in a real‐world cohort. No deaths as a result of failed shocks were discovered, which may reflect selection and survivor bias. The true incidence of failed shocks may be underreported and likely varies depending on the patient cohort.

The optimal management of failed shocks remains unclear from this retrospective report. The small sample size limits the ability to detect differences between management strategies or adjust for potential demographic differences. Increased sympathetic tone from acute illness, decompensated heart failure, repetitive arrhythmias, et cetera, negatively impacts defibrillation thresholds [3] and may result in failed shocks. In such cases, addressing the underlaying cause alone may be appropriate; though many patients underwent additional management and repeat defibrillation testing, resulting in a low rate of repeat events. Randomized controlled trials have shown that routine defibrillation testing may not necessary for native device implants [4]. Less is known if testing is required in the case of failed shocks (with or without invasive management) and should be the focus of future prospective study. Nevertheless, this strategy led to overall low rates of repeat failed shocks and should be considered pending patient candidacy. Adding additional transvenous or subcutaneous leads can attain more favorable shocking vectors increasing the likelihood of successful defibrillation; these invasive approaches must be assessed in the context of a patients procedural candidacy including vascular access and infectious disease concerns. Even with invasive management, repeat failed shocks were common highlighting the limited prognostic value of defibrillation testing and the complex and dynamic substrate of these patients.

The findings of a high rate of repeat failed shocks (particularly among patients without clear reversible causes who underwent procedural management) highlights the need for further innovation in device technologies as well as the importance of aggressively pursuing rhythm control in these high‐risk patients. Further multicenter studies with detailed reporting standards are needed to better understand the optimal management strategies of failed shocks.

## Conflicts of Interest

The authors declare no conflicts of interest.
